# ATP competes with PIP_2_ for binding to gelsolin

**DOI:** 10.1371/journal.pone.0201826

**Published:** 2018-08-07

**Authors:** Dávid Szatmári, Bo Xue, Balakrishnan Kannan, Leslie D. Burtnick, Beáta Bugyi, Miklós Nyitrai, Robert C. Robinson

**Affiliations:** 1 Institute of Molecular and Cell Biology, A*STAR (Agency for Science, Technology and Research), Singapore, Singapore; 2 University of Pécs, Medical School, Department of Biophysics, Pécs, Hungary; 3 Department of Biochemistry, National University of Singapore, Singapore, Singapore; 4 NUS Synthetic Biology for Clinical and Technological Innovation (SynCTI), Life Sciences Institute, National University of Singapore, Singapore, Singapore; 5 Department of Chemistry and Centre for Blood Research, Life Sciences Institute, University of British Columbia, Vancouver, BC, Canada; 6 Szentágothai Research Center, Pécs, Hungary; 7 Research Institute for Interdisciplinary Science, Okayama University, Okayama, Japan; Russian Academy of Medical Sciences, RUSSIAN FEDERATION

## Abstract

Gelsolin is a severing and capping protein that targets filamentous actin and regulates filament lengths near plasma membranes, contributing to cell movement and plasma membrane morphology. Gelsolin binds to the plasma membrane via phosphatidylinositol 4,5-bisphosphate (PIP_2_) in a state that cannot cap F-actin, and gelsolin-capped actin filaments are uncapped by PIP_2_ leading to filament elongation. The process by which gelsolin is removed from PIP_2_ at the plasma membrane is currently unknown. Gelsolin also binds ATP with unknown function. Here we characterize the role of ATP on PIP_2_-gelsolin complex dynamics. Fluorophore-labeled PIP_2_ and ATP were used to study their interactions with gelsolin using steady-state fluorescence anisotropy, and Alexa488-labeled gelsolin was utilized to reconstitute the regulation of gelsolin binding to PIP_2_-containing phospholipid vesicles by ATP. Under physiological salt conditions ATP competes with PIP_2_ for binding to gelsolin, while calcium causes the release of ATP from gelsolin. These data suggest a cycle for gelsolin activity. Firstly, calcium activates ATP-bound gelsolin allowing it to sever and cap F-actin. Secondly, PIP_2_-binding removes the gelsolin cap from F-actin at low calcium levels, leading to filament elongation. Finally, ATP competes with PIP_2_ to release the calcium-free ATP-bound gelsolin, allowing it to undergo a further round of severing.

## Introduction

Gelsolin plays important roles in the dynamics and regulation of actin filament lengths [1]. Calcium ions activate gelsolin to sever actin filaments, leading to one of the two resulting filaments being capped at its barbed end. Cellular gelsolin is mostly in the cytoplasm where F-actin severing, capping and uncapping by gelsolin, close to the plasma membrane, are thought to contribute to cell movement and membrane morphology [2–4]. Severed actin filaments, which are capped by gelsolin at their barbed ends, can be uncapped to produce directed polymerization at the plasma membrane. Phosphatidylinositides are involved in signaling to the actin cytoskeleton by modifying the activity of various actin-binding proteins, including the gelsolin superfamily proteins [5]. In particular, phosphatidylinositol 4,5-bisphosphate (PIP_2_) is a major regulator of actin cytoskeletal organization [6, 7] that modulates many actin regulating proteins [8], including: actin filament capping proteins, such as gelsolin [9], CapG [10] and capping protein [11]; actin monomer-binding proteins, like profilin [12], cofilin [13] and twinfilin [14]; actin filament nucleation effectors, including WASP [15], N-WASP [16] and dynamin2/cortactin [17]; actin filament crosslinking proteins, exemplified by α-actinin [18], filamin [19] and cortexillin [20]; and actin filament plasma membrane tethering proteins, represented by vinculin [21], talin [21] and ERM proteins [22]. When gelsolin localizes to PIP_2_-rich areas of the plasma membrane [23], PIP_2_ inhibits interactions between membrane-bound gelsolin and actin [9, 24–27] and removes gelsolin caps from actin filaments [9]. There is strong evidence to suggest that local accumulation of PIP_2_ at the plasma membrane leads to the uncapping of gelsolin-capped filaments, resulting in rapid, directed filament elongation [9, 28].

Three PIP_2_-binding sites have been identified on gelsolin: between residues 135–142, which overlaps with one of the G-actin binding sites; between residues 161–169, which overlaps with the F-actin binding site; and between residues 621–634, which overlaps with the ATP binding site [27, 29–31]. The second site is well conserved within the gelsolin superfamily. The mechanism of uncapping is not fully understood, however PIP_2_ may either directly compete with actin for binding to gelsolin, and/or it may change the conformation of the actin-binding sites to become incompatible with binding to actin [26, 31–33]. There are several reports of a correlation between PIP_2_ and calcium binding to gelsolin, however it is controversial whether this correlation is positive or negative [27, 29, 34].

Gelsolin can bind to the ATP-mimetic resin Cibracon-Blue and can be liberated from the resin by a range of nucleotides including ATP, ADP, GTP and GDP [35, 36]. Equilibrium dialysis experiments were used to determine dissociation constants of 0.28 μM and 1.8 μM for gelsolin with ATP and GTP, respectively, at high NaCl concentrations, while ADP and GDP showed no significant association under these conditions [37]. The affinity of ATP for gelsolin decreases (*Kd* = 2.4 μM) in the presence of 0.2–2.0 mM Mg^2+^ (pMg 4–3, where pMg = —log[Mg^2+^]). No ATP binding to gelsolin is detectable in solutions that contain more than 10 μM Ca^2+^ (pCa 5, where pCa = —log[Ca^2+^]) and conversely the presence of ATP reduces the affinity of gelsolin for Ca^2+^ [38]. Gremm and Wegner reported a lower affinity of calcium-free gelsolin for ATP (*Kd* = 32 μM) than discussed above [38]. This may reflect differences in analytical techniques, fluorescence analysis versus equilibrium dialysis, or differences in experimental buffer conditions, including pH, MgCl_2_ and CaCl_2_. Gelsolin does not show any detectable ATPase activity [37, 39]. The discovery of the gelsolin:ATP interaction led to the suggestion that ATP may be important in some of the multiple functions of gelsolin. The structure of the gelsolin:ATP complex revealed the basis for its sensitivity to calcium ion concentrations in that ATP interacts with both of the two halves of calcium-free gelsolin, which change conformation on binding to calcium [32, 40–45]. Thus, the loss of its ATP-binding ability is due to disruption of the interaction site within gelsolin caused by a conformational change in response to calcium [37]. The phosphate groups of ATP interact with basic residues on gelsolin domain 5 (G5, residues 514–619). These residues also comprise part of a region that previously had been determined to bind to PIP_2_ [27]. Gelsolin is also sensitive to the type of nucleotide bound to actin; it severs filaments that contain ADP-actin but not ADP-Pi-actin units [46]. Accordingly, G4-G6 (residues 418–741) shows a preference for ADP-containing actin monomers while G1-G3 (residues 25–370) binds to ATP- and ADP-actin with comparable affinities [47]. In contrast, ATP (but not ADP) concentrations in the mM range inhibit the binding of G1-G3 to actin monomers [47].

Generally, the free calcium concentrations in resting cells oscillate on the nanomolar scale [48, 49], however in stimulated cells these concentrations can increase to micromolar levels [50, 51]. Cytoplasmic free magnesium levels are regulated in the pMg 4–3 (0.5–1 mM) range [52, 53]. Intracellular ATP concentrations (2 μM—8 mM, mean value 0.5–1 mM) can change over a wide range depending on the type, stage and state of the cell, and these levels play a regulatory role in plasma membrane channel function [54–61]. Gelsolin is activated by calcium, not by magnesium, to sever and cap filaments. However the gelsolin/actin complex is not dissociated by the lowering of calcium levels, with one bound calcium ion becoming trapped in the complex [62]. In this report we probe the interplay between ATP and calcium in modifying PIP_2_ binding to gelsolin. We postulate, based on our *in vitro* data, which ATP plays a critical role in the recycling of gelsolin by removing gelsolin from PIP_2_ in the plasma membrane, leading to the release of gelsolin into the cytoplasm to undergo further rounds of calcium-induced actin filament severing.

## Experimental procedures

### Proteins

His-tagged human wild-type gelsolin was expressed in *E*. *coli* strain Rosetta2 (DE3) pLysS cells from a pSY5 plasmid [45]. The protein was subjected to Ni-NTA affinity chromatography, HRV 3C protease cleavage, followed by gel filtration (Superdex 200, GE Healthcare) in 10 mM Tris-HCl, 150 mM NaCl, pH 8. Traces of calcium were removed by dialysis (2 mM Tris-HCl, 1 mM EGTA, pH 7.4, overnight). Rabbit skeletal muscle actin was prepared from acetone powder (Pel-Freez Biologicals) in a protocol modified from Spudich and Watt [63, 64]. Actin was stored in buffer A (2 mM Tris-HCl, 0.2 mM ATP (ATP disodium trihydrate, Sigma-Aldrich), 0.1 mM CaCl_2_ (pCa 4), 0.1 mM DTT and 0.005% NaN_3_, pH 7.4). Alexa Fluor^TM^ 488 C5 Maleimide (Life Technologies) was used to label cysteine residues of gelsolin based on the manufacturer’s recommendations (Thermo Fisher Scientific).

### Phospholipid vesicle preparation

Phospholipid vesicles were prepared by a modified protocol from James H. Morrissey, Dept. of Biochemistry, University of Illinois at Urbana-Champaign, Urbana, IL 61801, USA (https://tf7.org). A mixture of 1% PIP_2_ (PtdIns-(4,5)-P_2_(1,2-dipalmitoyl)) (Cayman Chemicals), 79% PC (L-α-phosphatidylcholine) (Sigma-Aldrich) and 20% PS (3-sn-phosphatidyl-L-serine) (Sigma-Aldrich) was dissolved in 20 μM rhodamine 590 N-succinimidyl ester (Sigma-Aldrich) in chloroform. The dried lipid mixture was sonicated in an Elmasonic S100H water bath sonicator (medium strength of pulses) in 2 mM Tris-HCl, 200 mM NaCl, pH 7.4, with the temperature maintained at 40 ^o^C, until the solution became visually homogeneous (approx. 30 mins) and small multilamellar vesicles were formed. Vesicles were collected by centrifugation at 22°C for 10 min at 5,000 x g, and stored at 4°C.

### Gelsolin intrinsic tryptophan fluorescence

The intrinsic tryptophan (residue numbers: 67, 111, 203, 223, 341, 392, 446, 489, 601, 637, 699, 706, 755, 759, 764) fluorescence emission assays were carried out with a Perkin Elmer LS-50 spectrofluorimeter. The excitation and emission monochromators were set to 288 nm and 332 nm, respectively, and the excitation and emission bandwidths to 5 nm. 5 μM gelsolin was incubated under physiological salt conditions (100 μM CaCl_2_ (pCa 4), 100 mM KCl, 1 mM MgCl_2_ (pMg 3), 0.2 mM ATP, 2 mM Tris-HCl, pH 7.4) supplemented with EGTA or CaCl_2_ to vary the free calcium levels (calculated with Maxchelator Stanford http://maxchelator.stanford.edu/CaMgATPEGTA-NIST.htm): pCa 9: 6 mM EGTA; pCa 6: 100 μM EGTA; pCa 3: 1 mM CaCl_2_. Steady-state fluorescence intensities were measured after sequential addition of appropriate stock solutions to attain: **1)** 2 μM PIP_2_, **2)** 0.5 mM ATP, and **3)** pCa 6.

### Steady-state fluorescence anisotropy

We used a fluorescent derivative of PIP_2_ (PtdIns-(4,5)-P_2_-fluorescein, Cayman Chemicals, NU-10010388, λ_ex_ = 493 nm, λ_em_ = 520 nm, abbreviated as PIP_2_-F) and a fluorescent derivative of ATP (N^6^-(6-amino)hexyl-ATP-ATTO-532, Jena Bioscience, NU-805-532, λ_ex_ = 532 nm, λ_em_ = 553 nm, abbreviated as ATP-N) as probes for the binding of PIP_2_ and ATP to gelsolin. Fluorescence intensities were measured on a Safire^2^ monochromator microplate reader (TECAN) at 22°C. Steady-state fluorescence anisotropy values were determined according to Eq 1 [65], where I_VV_ and I_VH_, respectively, are the intensities of vertically and horizontally polarized emission on excitation with vertically polarized light, with the correction factor, G, being the ratio of I_VH_/I_VV_.

$$r=\frac{I_{VV}-GI_{VH}}{I_{VV}+2GI_{VH}}$$ (1)

The dissociation constants for ATP and PIP_2_ were calculated using a hyperbolic model [66] from anisotropy data that had reached saturation during the gelsolin titrations Eq 2:

$$r=r_{f}+(r_{b}-r_{f})\;\left(\frac{K_{d}+\left[G\right]+\left[L\right]-\sqrt{{\left(K_{d}+[G]+[L]\right)}^{2}-4[G][L]}}{2[L]}\right)$$ (2)

where *r*_*f*_ is the anisotropy of free ligand, *r*_*b*_ is the anisotropy of gelsolin-bound ligand, *[L]* is the total concentration of ligand, *Kd* is the dissociation constant and *[G]* is the total concentration of gelsolin. The anisotropy of 0.5 μM ATP-N was measured in the presence of gelsolin concentrations: 0, 0.2, 1.5, 2, 3, 5 and 8 μM. Saturation was evident at 5 μM characterized by an anisotropy value of 0.15 ± 0.006. The calcium and magnesium sensitivities of ATP binding to gelsolin were characterized by the change in anisotropy of solutions containing 0.5 μM ATP-N in the presence of 5 μM of gelsolin over a range of divalent cation concentrations of pCa 2–12 or pMg 1–12, where pCa and pMg refer to, respectively. The affinity of the PIP_2_-gelsolin interaction was measured by the anisotropy change of 0.5 μM or 0.25 μM PIP_2_-F in the presence of 0, 0.2, 2, 1.5, 3, 5 and 8 μM gelsolin, which showed saturation at 4 μM. Anisotropy of 0.5 μM or 0.25 μM solutions of PIP_2_-F in the presence of 5 μM gelsolin was used to probe the changes in binding of the complexes under different calcium, magnesium, salt and ATP conditions.

### Confocal microscopy imaging

10% (v/v) rhodamine590-filled phospholipid vesicles and 5 μM Alexa488-labeled gelsolin were incubated together and a drop of the mixture was placed on a clean glass slide, which was then covered by and separated from a second slide by a parafilm gasket. In this setup, buffer conditions surrounding the phospholipid vesicles can be changed by laminar flow between the two slides. A 2 mm x 2 mm piece of tissue (KimWipes) was used to secure the vesicles. The sample was washed (2 mM Tris-HCl, pH 7.4) for 1 min and then placed on the glass slide before being covered. In the flow cell, phospholipid vesicles were kept in the field of view by the tissue fibers. Fluorescence emission-based imaging was carried out using a Zeiss LSM 510 META Confocal Microscope. The two different fluorophores were detected in two separate channels; Alexa488 (gelsolin) was excited by 477 nm laser light and emission was detected in the 505–530 nm channel, while rhodamine590 (phospholipid vesicles) was excited by 545 nm laser light and emission was detected in the 585–630 nm channel.

### Statistics

The data presented were derived from at least 3 independent experiments. Values are displayed as the mean ± standard deviation.

## Results

**Fig 1:**
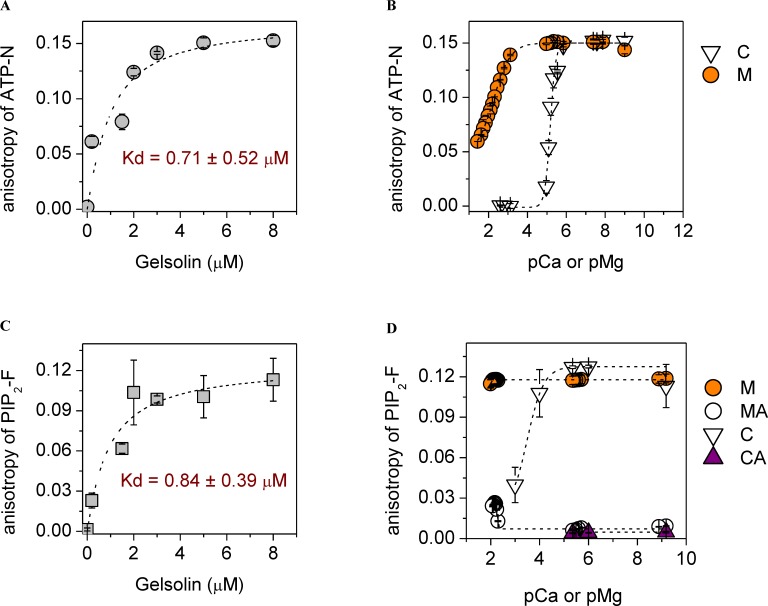
The interplay between calcium, magnesium, ATP and PIP_2_ for binding to gelsolin. **(a)** In the absence of cations, the anisotropy of ATP-N (0.5 μM) increased and saturated by ~ 5 μM gelsolin. Dashed line shows the fit to the data (Eq 2). **(b)** Micromolar calcium levels (C, open triangles, inflection point at pCa 5.2) or millimolar magnesium levels (M, orange circles, inflection point at pMg 2.3) were able to reduce the anisotropy of ATP-N (0.5 μM) in the presence of gelsolin (5 μM), indicating the dissociation of ATP-N from gelsolin. Dashed line indicates sigmoidal fitting. **(c)** In the absence of cations, the anisotropy of PIP_2_-F (0.5 μM) increased and reached steady-state at ~ 5 μM gelsolin, indicating saturation of binding. Dashed line shows the fit to the data (Eq 2). **(d)** The anisotropy of PIP_2_-F (0.5 μM) in the presence of gelsolin (5 μM) was not changed on titration with magnesium (M, orange circles), but inclusion of 0.5 mM ATP (MA, white circles) lowered the anisotropy to the value characteristic to free PIP_2_-F, indicating complete dissociation of the gelsolin/PIP_2_-F complex by ATP. This effect was diminished by magnesium concentrations above 7 mM (pMg 2.15). In the absence of ATP, PIP_2_-F (0.5 μM) binds to gelsolin (5 μM), as revealed by the increase in anisotropy across a wide range of calcium concentrations (C, white triangles). Inclusion of ATP (0.2 mM, CA, purple triangles) lowered the anisotropy to the value characteristic to free PIP_2_-F, indicating complete dissociation of the gelsolin/PIP_2_-F complex by ATP.

In order to probe the interplay between PIP_2_, ATP and Ca^2+^ in binding to gelsolin, we first characterized the binding of fluorescent derivatives of PIP_2_ and ATP to gelsolin using steady-state anisotropy measurements. The fluorescent derivative of ATP (0.5 μM), N^6^-(6-amino)hexyl-ATP-ATTO-532, named ATP-N hereafter, binds to gelsolin with a *Kd* of 0.71 ± 0.52 μM in the absence of divalent cations, as determined by analysis of steady-state anisotropy measurements (Fig 1*A*). This value is within experimental error of that previously reported for the gelsolin:ATP interaction (0.28 μM), as measured by equilibrium dialysis [39]. Micromolar levels of calcium (pCa 6) ions or millimolar levels of magnesium (pMg 3) ions were able to effectively dissociate ATP-N from gelsolin (5 μM), characterized by 50% of the ATP-N remaining bound at 6.3 ± 0.2 μM calcium or 5.0 ± 1.1 mM magnesium (Fig 1*B*). This calcium range that induces ATP dissociation is in line with physiological calcium signaling levels and with calcium concentrations that are able to initiate conformational changes in gelsolin, which disrupt the ATP-binding site leading to ATP release [42]. The magnesium levels that release ATP-N from gelsolin are close to the reported affinity of ATP for Mg^2+^ [67], which is in line with gelsolin/ATP structure [32], which does not contain cations. Thus, the Mg^2+^-induced release of ATP from gelsolin by Mg^2+^ alone is likely due the incompatibility of Mg-ATP with gelsolin. However, the effective concentration of magnesium is higher than normal free physiological levels pMg 4–3 (0.5–1.0 mM) [52]. We next tested the effect of ionic strength by the addition of potassium ions, which weakened the gelsolin:ATP-N interaction (S1 Fig). Under physiological potassium ion concentrations, the ATP-N interaction with gelsolin was characterized by *Kd*s of 1.27 ± 0.60 μM and 1.42 ± 0.27 μM in 100 mM and 120 mM KCl, respectively, values which were increased slightly by the addition of 1 mM MgCl_2_ (pMg 3) (*Kd*s of 1.58 ± 0.85 μM and 2.34 ± 0.73 μM, respectively) (S1 Fig). Similarly, in 50 mM KCl and 1 mM MgCl_2_ (pMg 3), a condition commonly used for *in vitro* actin polymerization experiments [63], gelsolin binding of ATP-N was determined by titration with gelsolin and via competition of ATP-N binding by unlabeled ATP, and the *Kd* values were 1.8 μM (ATP-N) and 2.2 μM (ATP) (S2 Fig). The effect of calcium under these actin polymerization conditions recapitulated the observation made under the low salt conditions, with micromolar (pCa 6) and higher concentrations of calcium negatively impacting the gelsolin:ATP-N interaction (Panel *a* in S2 Fig vs. S1 Fig). Collectively, these data indicate that under physiological buffer conditions gelsolin binds to both ATP-N and unlabeled ATP in similar fashion and that calcium acts as a regulator for the binding.

Gelsolin has been shown to have three PIP_2_-binding sites, which have previously been characterized to display *Kd* values in the 1–20 μM range [27, 29, 31]. In a similar strategy to that adopted for characterizing the ATP-gelsolin interaction, a soluble fluorescent derivative of PIP_2_, PtdIns-(4,5)-P_2_-fluorescein, named PIP_2_-F hereafter, was used to probe the gelsolin-PIP_2_ interaction using steady-state anisotropy measurements. In this assay the binding of PIP_2_-F (0.5 μM) to gelsolin in the absence of divalent cations was characterized by a *Kd* of 0.84 ± 0.39 μM (Fig 1*C*). This value increased to 1.35 ± 0.7 μM and 3.12 ± 1.7 μM in the presence of increased ionic strength, 100 mM and 120 mM KCl, respectively, and these values remained constant within experimental error on the further addition of 1 mM MgCl_2_ (pMg 3) (S3 Fig). Thus, the affinities of gelsolin for PIP_2_-F and ATP-N lie in the same range under physiological salt conditions.

Subsequently, we studied the sensitivity of PIP_2_-F (0.5 μM) binding to gelsolin (5 μM) in the absence/presence of ATP and divalent cations (magnesium and calcium). It is known that divalent cations, calcium in particular, cause PIP_2_ to aggregate [68, 69]. Therefore, we evaluated the effects of cations on the critical micelle concentrations (CMCs) of PIP_2_ and PIP_2_-F by dynamic light scattering [70]. Under the buffer conditions tested, PIP_2_-F failed to form micelles at or below concentrations of 0.5 μM, whereas PIP_2_ was more sensitive to cations and had a greater tendency to form micelles (S4 Fig). Thus, we used 0.5 μM PIP_2_-F to probe its interactions with gelsolin.

**Fig 2:**
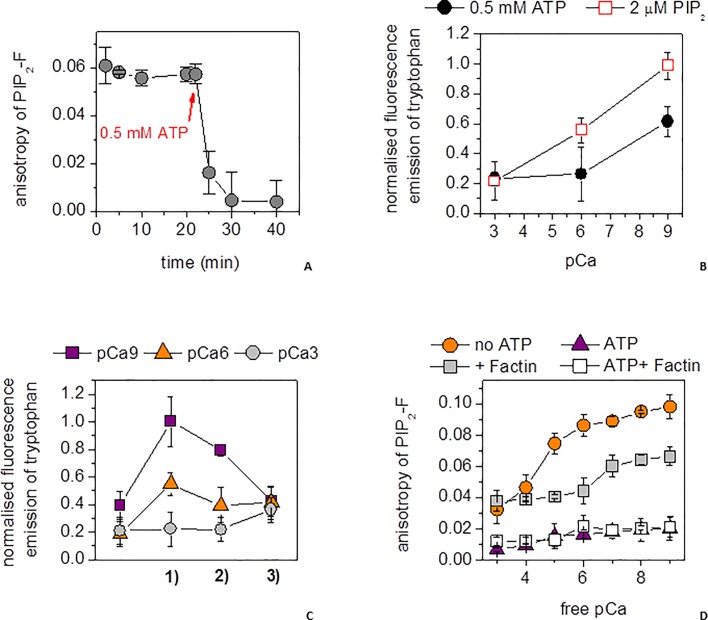
PIP_2_ binding to gelsolin under actin polymerizing conditions. **(a)** The anisotropy of PIP_2_-F (0.25 μM) was measured as a function of time in the presence of 5 μM gelsolin, 10 μM Ca^2+^(pCa 5), 1 mM Mg^2+^ (pMg 3) and 50 mM K^+^ (gray circles). After 20 min, ATP (0.5 mM) was added. **(b)** Tryptophan fluorescence emission of gelsolin (5 μM) was measured at steady-state at three free-calcium concentrations: 1 nM (pCa 9), 1 μM (pCa 6) and 1 mM (pCa 3) in the presence of 2 μM PIP_2_ (red open squares) or 0.5 mM ATP (filled black circles). **(c)** Tryptophan fluorescence emission of gelsolin (5 μM) was measured at steady-state at three free-calcium concentrations: 1 nM (pCa 9, purple squares), 1 μM (pCa 6, orange triangles) and 1 mM (pCa 3, gray circles) through a cycle of **1)** 2 μM PIP_2_ addition, **2)** 0.5 mM ATP addition and **3)** calcium levels set to 1 μM (pCa 6). **(d)** The steady-state anisotropy of PIP_2_-F (0.25 μM) in the presence of gelsolin (5 μM) under actin polymerizing salt conditions (1 mM Mg^2+^ (pMg 3), 100 mM K^+^, orange circles) followed a similar profile to that without salt across a wide range of calcium ion concentrations (see data presented on Fig 1*D*). Addition of F-actin (50 μM, gray squares) reduced the anisotropy, but still indicated significant binding. Inclusion of ATP (0.5 mM) reduced the anisotropy close to background levels, to the value characteristic to free PIP_2_-F both in the presence (white squares) and absence (purple triangles) of F-actin (50 μM) indicating the dissociation of the gelsolin/PIP_2_-F complex across the entire calcium concentration range.

The first evidence of the competition between ATP and PIP_2_ in binding to gelsolin was observed from the elevated anisotropy (0.12, Fig 1*C*) of PIP_2_-F (0.5 μM), characteristic of its association with gelsolin (5 μM), being lost on the addition of 0.2 or 0.5 mM ATP (Fig 1*D*). The ability of ATP to dissociate PIP_2_-F remained constant over a wide range of Mg^2+^ concentrations, from picomolar (pMg 12) to the physiologically relevant millimolar (pMg 3) range, becoming less effective above 7 mM (pMg 2.15). Hence, ATP can effectively compete with PIP_2_ for binding to gelsolin under physiological Mg^2+^ conditions. In the absence of ATP, PIP_2_-F was able to bind gelsolin, characterized by a steady-state anisotropy value of approximately 0.12, over the ≈0–10 μM range of Ca^2+^ (pCa 15–5)(Fig 1*D*). At higher calcium levels the anisotropy began to reduce, reaching 0.04 at 1 mM Ca^2+^ (pCa 3)(Fig 1*D*). The PIP_2_-F/gelsolin interaction was characterized by *Kd*s below 20 μM across the entire calcium range, in line with previously reported PIP_2_/gelsolin affinities [27, 29, 31], and below 1 μM in the 1–10 μM range (pCa 6–5) (Panel *a* in S5 Fig), in the absence of KCl and magnesium. In the presence of 0.2 mM ATP in the ≈0–10 μM calcium (pCa 15–5) range, the anisotropy was close to the baseline characteristic of free PIP_2_-F, indicating the effective dissociation of the PIP_2_-F/gelsolin interaction (Fig 1*D*) (Panel *a* in S5 Fig) with estimated *Kd*s in excess of 300 μM (Panel *a* in S5 Fig). Higher calcium levels were not obtainable in the presence of ATP due to precipitation of these reagents in the absence of salt (Panel *b* in S5 Fig). In a control experiment, PIP_2_ had no observable effect on the binding of calcium to FURA-2FF, implying that any calcium binding by PIP_2_ is substantially weaker than that of FURA-2FF (*Kd* = 25 μM, Panel *c* in S5 Fig), and it is unlikely to have had a significant effect in these experiments. Thus, these data suggest that ATP can release PIP_2_-F from gelsolin effectively in the ≈0–10 μM calcium (pCa 15–5) concentration range. We confirmed that the PIP_2_-F-gelsolin complex remained intact under actin polymerization conditions, and that PIP_2_-F was released from gelsolin when these conditions were supplemented with 0.5 mM ATP (Fig 2*A*), the calculated K*d*s ranged from 2.7 ± 0.2 μM in absence of ATP followed by a substantial rise to 65.4 ± 6.2 μM after the addition of ATP.

Subsequently, we used the intrinsic fluorescence emission at 332 nm from the 15 tryptophan residues evenly distributed across the 6 domains of gelsolin to probe the effects of higher concentrations of PIP_2_ and ATP on gelsolin than could be probed by the PIP_2_-F and ATP-N anisotropy assays. Since gelsolin contains 6 calcium-binding sites, some of which have *Kd* values below the protein concentration used in the experiment, we turned to an EGTA-buffered calcium system to control the free calcium levels. Gelsolin was incubated under actin polymerizing conditions (100 μM CaCl_2_ (pCa 4), 100 mM KCl, 1 mM MgCl_2_ (pMg 3), 0.2 mM ATP, 2 mM Tris-HCl, pH 7.4), which was supplemented with EGTA or CaCl_2_ to vary the free calcium levels. Gelsolin (5 μM) showed lower tryptophan emission levels when bound to ATP (0.5 mM) relative to PIP_2_ (2 μM) at 1 μM (pCa 6) and 1 nM (pCa 9) CaCl_2_ (Fig 2*B*). This difference was used to demonstrate the cycling between gelsolin binding to PIP_2_ and ATP. Gelsolin showed a decrease in tryptophan fluorescence in moving from 1 nM (pCa 9) free calcium to activating levels of calcium (pCa 6 and pCa 3, Fig 2*C*). Subsequent addition of PIP_2_ (2 μM) to these conditions raised the intrinsic tryptophan fluorescence at 1 nM (pCa 9) and 1 μM (pCa 6), but not at 1 mM (pCa 3) free calcium, indicating that tryptophan fluorescence is a sensitive reporter on the gelsolin/PIP_2_ interaction in the pCa 9 to pCa 6 range (Fig 2*C*/1). Subsequent addition of ATP (0.5 mM) led to a reduction in the intrinsic fluorescence at both pCa 9 and pCa 6 free calcium (Fig 2*C*/2). Finally, the tryptophan fluorescence converged on adjusting each condition to pCa 6 free calcium, indicating that the interplay between calcium and gelsolin in PIP_2_/ATP was in equilibrium and reversible (Fig 2*C*/3). Thus, the tryptophan assay shows that ATP can exert competitive effects against PIP_2_ in the pCa 9 and pCa 6 calcium range.

Next we probed the effect of actin on the competition between ATP and PIP_2_ in binding to gelsolin. The association of PIP_2_-F (0.25 μM) with gelsolin (5 μM) was investigated as a function of free Ca^2+^ concentration under actin polymerizing salt conditions (100 mM K^+^, 1 mM Mg^2+^ (pMg 3)). Addition of 0.5 mM ATP caused an almost complete loss of PIP_2_-F anisotropy across the entire calcium range, both in the absence and presence of 50 μM actin (Fig 2*D*). Together these data indicate that ATP can release PIP_2_ from gelsolin in the presence of salt, calcium ions and actin.

**Fig 3:**
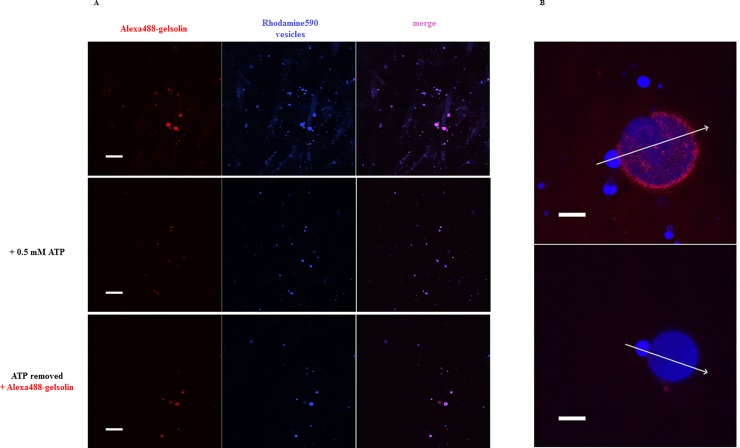
ATP dependence of gelsolin binding to the surface of PIP_2_-containing membrane vesicles. **(a)** Gelsolin-Alexa488 (5 μM, red) was incubated with rhodamine590-filled, PIP_2_-containing membrane vesicles (blue) in the absence of divalent cations and visualized by confocal microscopy. The merged image indicates that gelsolin and vesicles colocalized (top panel). After ATP (0.5 mM) was added, the majority of gelsolin-Alexa488 was released from the vesicles (middle panel). Following removal of ATP via buffer exchange and addition of fresh gelsolin-Alexa488 (15 μM), gelsolin re-associated with the vesicles (bottom panel). Scale bar = 10 μm. S1 Movie details the time course of these changes. **(b)** Representative image of gelsolin-Alexa488 (15 μM, red) localized to the surface of a large rhodamine590-filled PIP_2_-containing vesicle (blue) (top panel). After the addition of ATP (0.5 mM) the vesicle changed morphology concurrently with the release of gelsolin (red) from the vesicle surface (blue) (bottom panel). Scale bar = 10 μm. Panel *a* and *b* in S6 Fig show line scans across the images as indicated by the arrows and S2 Movie details the time course. The confocal slice thickness was ~ 3 μm. There are two vesicles in focus, a small (d = 7 μm) and a large (d = 22.5 μm) one, the outer layer of the bigger vesicle is attached to the coverslip.

Finally, we used confocal microscopy to determine whether ATP can be observed to release gelsolin from phospholipid vesicles. PIP_2_-containing rhodamine590-filled phospholipid vesicles were observed to bind to Alexa488-labeled gelsolin in the absence of ATP, calcium and magnesium (S1 Movie; Fig 3*A*, upper panel). This gelsolin was subsequently released by the addition of 0.5 mM ATP (S1 Movie; Fig 3*A*, middle panel). A second round of labeled gelsolin could be bound to the phospholipid vesicles after the solution had been exchanged to remove the ATP (S1 Movie; Fig 3*A*, lower panel). During the release of gelsolin by ATP, some phospholipid vesicles were observed to display shape changes (S2 Movie; Fig 3*B*, S6 Fig). This suggests that gelsolin binds to PIP_2_ in the phospholipid vesicles and may induce deformation or local structural changes in the vesicles and that ATP can effectively dissociate gelsolin from the phospholipid vesicles.

## Discussion

ATP and PIP_2_ have been shown to compete in binding to K-ATP channels [71]. Here, we have demonstrated that ATP can displace PIP_2_ from gelsolin in solution under actin polymerizing buffer conditions *in vitro*. Furthermore, ATP is able to release PIP_2_-bound gelsolin from the surface of phospholipid vesicles. These observations suggest that ATP is likely to dissociate gelsolin from PIP_2_ at plasma membranes, and this ATP-driven dissociation is the missing step in recycling of gelsolin during its actin filament remodeling cycle. In a background of high cellular ATP, PIP_2_ will not generally bind to gelsolin. However, in the situation where gelsolin-capped filaments point at the plasma membrane, the filament barbed-end bound gelsolin becomes greatly reduced in its mobility, and it is in close proximity to membrane-bound PIP_2_ that can move within the plasma membrane and increase its local concentration by forming clusters [72]. All these factors favor the binding between gelsolin and PIP_2_, and hence, filament uncapping. We propose that effective competition by ATP will dominate following filament uncapping and that diffusion of PIP_2_-bound gelsolin will increase.

**Fig 4:**
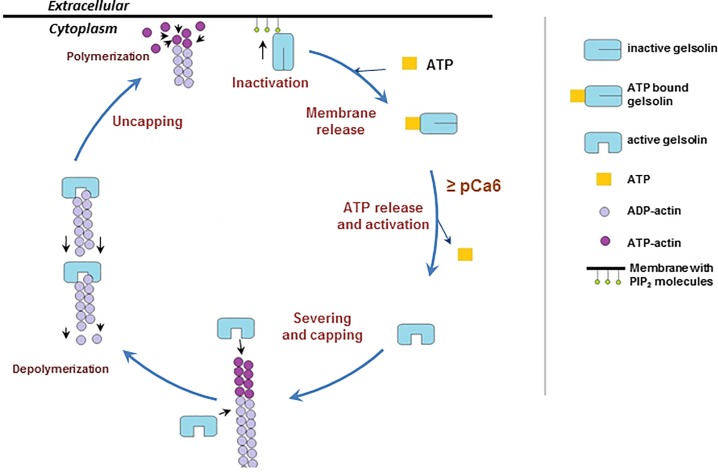
Model of the severing, capping, uncapping and inactivation/release cycle of gelsolin. The cartoon presents a model for the cycle of activation and function of gelsolin. Severing and capping: Elevated free calcium levels activate gelsolin, releasing ATP, leading to severing and capping of actin filaments. Depolymerization: Gelsolin-capped filaments will depolymerize from their pointed ends. Uncapping and polymerization: Gelsolin-capped actin filaments will be uncapped on encountering PIP_2_ in the membrane, resulting in force being exerted on the membrane from the polymerization of the uncapped filaments. Inactivation and membrane release: Gelsolin will be released from PIP_2_ and the membrane by competition with ATP. Following its release gelsolin is able to undergo subsequent cycles of severing, capping, uncapping and inactivation/release.

The cartoon presented in Fig 4 details the postulated stages of this remodeling cycle under standard cellular conditions. Activation: elevation of calcium levels leads to a conformational change in gelsolin that releases ATP and allows gelsolin to recognize an actin filament. Severing: competition for actin-actin interactions by gelsolin-actin interactions leads to the severing of the filament. Capping: gelsolin remains bound to the barbed-end of the severed filament, preventing its elongation. Uncapping: when a gelsolin-capped filament encounters PIP_2_ in the plasma membrane, the cap is removed through an unknown mechanism. The uncapped filament is then free to elongate and exert force on the plasma membrane. Release: gelsolin is released from PIP_2_ at the plasma membrane through ATP competition, leading to diffusion of the gelsolin:ATP complex away from the plasma membrane. Gelsolin will return to its inactive state in low calcium environments. Thus, gelsolin is likely removed from PIP_2_ at the plasma membrane in an ATP-dependent manner that distinguishes it from other PIP_2_-sensing actin-regulating proteins, allowing gelsolin to cycle in a background of elevated PIP_2_.

## Supporting information

S1 Fig The interplay among calcium, magnesium and potassium ions on ATP-N binding to gelsolin. In the absence of divalent cations, the anisotropy of ATP-N (0.5 μM) increased with increasing gelsolin concentration and this interaction was potassium ion dependent. The K*d*s calculated from the binding curves fitted with Eq 2 showed diminishing affinities with increasing potassium ion concentrations (blue triangles). Inclusion of 1 mM MgCl_2_ slightly weakened the affinity of ATP-N for gelsolin (magenta circles) at 100 mM and 120 mM KCl (K, potassium ions, KM, potassium and magnesium ions).
(PDF)

S2 Fig ATP binding to gelsolin under actin polymerization conditions. (A) The calcium-dependent gelsolin binding affinity for ATP under actin polymerization conditions was determined by monitoring the change in ATP-N anisotropy on gelsolin titration at different calcium ion concentrations. Each data point arises from the K*d* calculated from a titration similar to Fig 1*A*. K*d*s were calculated to be between 1.51 μM and 2.35 μM in the 1 nM to 10 μM calcium concentration range, and the value increased to 8.05 ± 1.32 μM above 100 μM calcium. (B) The anisotropy of ATP-N (0.5 μM) bound to gelsolin (5 μM) was measured upon titrating with unlabeled ATP in the absence of calcium. 50 μM ATP was sufficient to remove the gelsolin bound ATP-N. The affinity of unlabeled ATP for gelsolin, under actin polymerizing salt conditions, was calculated to be K*d* = 2.2 ± 0.17 μM measured by labeled/unlabeled ATP competition on gelsolin, which is similar to the affinity of ATP-N derived from Panel *c* in S1 Fig (K*d* = 1.58 ± 0.8 μM). Data were analyzed by the method of Kubala (Kubala et al. 2004) with the modification that the change in anisotropy of ATP-N was substituted for the change in fluorescence emission as the indication of ATP competition.
(PDF)

S3 Fig The interplay between calcium ions, magnesium ions and ionic strength on PIP_2_-F binding to gelsolin. In the absence of divalent cations, the anisotropy of PIP_2_-F (0.5 μM) increased with increasing gelsolin concentration and this interaction was potassium ion dependent. The K*d*s calculated from the binding curves fitted with Eq 2 showed diminishing affinities with increasing potassium ion concentrations (blue triangles). Inclusion of 1 mM MgCl_2_ slightly weakened the affinity of PIP_2_-F for gelsolin (magenta squares) at 100 mM and 120 mM KCl (K, potassium ions, KM, potassium and magnesium ions). (B) Anisotropy of PIP_2_-F (0.5 μM) in the presence of gelsolin (5 μM) as a function of KCl or NaCl concentration. Data were fitted with simple sigmoidal curves. The decrease in anisotropy indicates that PIP_2_-F is dissociated from gelsolin by increasing potassium or sodium ion concentrations, with the half-effective concentrations of 141.2 ± 3.0 mM and 143.3 ± 1.6 mM, respectively. This indicates the effect of KCl on gelsolin:PIP_2_-F binding is largely ionic and nonspecific.
(PDF)

S4 Fig Assessment of the solubility of PIP_2_ and PIP_2_-F in the experimental buffers. (A) Determination of the actual concentrations of PIP_2_-F after incubation with different cations by light absorbance at 494 nm. T, 2 mM Tris-HCl, pH 7.4; K, 100 mM KCl; C, 1 mM CaCl_2_; M, 1 mM MgCl_2_. (B) Determination of critical micelle concentrations of PIP_2_ and PIP_2_-F (Panel inset) by dynamic light scattering.
(PDF)

S5 Fig Assessment of the effects of calcium and ATP on the binding of ATP-N and PIP_2_-F to gelsolin. (A) K*d*s were calculated from the fit data with Eq 2 of gelsolin:PIP_2_-F binding under different calcium concentrations in the absence or presence of ATP. Part of the data have been shown in Fig 1*D* (white triangles). In the absence of ATP, PIP_2_-F (0.5 μM) binds to gelsolin, as reflected by thehigh affinity, across a wide range of calcium concentrations. In the presence of ATP, the interaction of PIP_2_-F with gelsolin was very weak below 10 μM calcium (K*d* = 563.0 ± 3.6 μM at 10 μM calcium). (B) In the absence of gelsolin, calcium directly precipitates ATP-N (C, open triangles) with a half-maximum value of 70.4 ± 0.7 μM. Magnesium has no effect on the fluorescence emission of ATP-N either in the absence (M, orange circles) or presence of 10 μM calcium (MC, purple squares). Data were fitted with simple sigmoidal curves. (C) The reported FURA-2FF dissociation constant for calcium ions is 25 μM (A.G. Scientific Inc.). The calcium-dependent fluorescence emission profile of FURA-2FF (2 μM) over pCa range of 5–7 was similar in the presence and absence of 20 μM PIP_2_, which were fitted with simple sigmoidal curves. The half-saturation pCa values were = 6.265 ± 0.004 and 6.217 ± 0.011 in the absence and presence of PIP_2_, respectively, suggesting that PIP_2_ does not interact with calcium with a K*d* less than 25 μM. The measurement was carried out with a Perkin Elmer LS-55 spectrofluorimeter (λ_ex_ = 340 nm, λ_em_ = 505 nm).
(PDF)

S6 Fig Line scans of fluorescence intensity versus distance along the arrows shown in Fig 3*B*. (A) Profiles of gelsolin-Alexa488 (red) and PIP_2_-containing vesicles filled with rhodamine590 (blue) in the absence of ATP. (B) Gelsolin-Alexa488 (red) was released and the size of phospholipid vesicle (filled by rhodamine590, blue) was changed by 0.5 mM ATP treatment in Fig 3*B*.
(PDF)

S1 Movie The influence of ATP on the binding of Alexa488-labeled gelsolin to PIP_2_-containing rhodamine590-filled phospholipid vesicles. Time course for Fig 3*A*. Gelsolin-Alexa488 (5 μM, red, top left) was incubated with rhodamine590-filled PIP_2_-containing membrane vesicles (blue, top right) and visualized by confocal microscopy. The merged image indicates that gelsolin and vesicles colocalized (bottom left). After ATP (0.5 mM) was added the majority of gelsolin-Alexa488 was released from the vesicles. Following removal of ATP via buffer exchange and addition of fresh gelsolin-Alexa488 (15 μM), gelsolin re-associated with the vesicles. The movie plays six times faster than the real time course.
(MP4)

S2 Movie Shape changes of phospholipid vesicles during the release of gelsolin by ATP. Time course for Fig 3*B*. After the addition of ATP (0.5 mM) the vesicle changed morphology concurrently with the release of gelsolin (red) from the vesicle surface (blue). The movie plays six times faster than the real time course.
(MP4)
