# Supplementary material for: ATP competes with PIP2 for binding to gelsolin
Source: PLoS One. 2018 Aug 7;13(8):e0201826. doi: 10.1371/journal.pone.0201826 (PMC6080781; doi:10.1371/journal.pone.0201826)
Supplement: S1 Fig — In the absence of divalent cations, the anisotropy of ATP-N (0.5 μM) increased with increasing gelsolin concentration and this interaction was potassium ion dependent. The Kds calculated from the binding curves fitted with Eq 2 showed diminishing affinities with increasing potassium ion concentrations (blue triangles). Inclusion of 1 mM MgCl2 slightly weakened the affinity of ATP-N for gelsolin (magenta circles) at 100 mM and 120 mM KCl (K, potassium ions, KM, potassium and magnesium ions). (PDF) [file pone.0201826.s001.pdf]

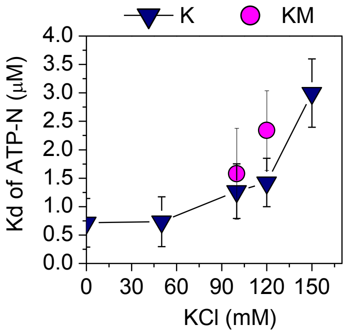

Fig. S1. The interplay among calcium, magnesium and potassium ions on ATP-N binding to gelsolin. In the absence of divalent cations, the anisotropy of ATP-N (0.5 μM) increased with increasing gelsolin concentration and this interaction was potassium ion dependent. The K*d*s calculated from the binding curves fitted with Eq. 2 showed diminishing affinities with increasing potassium ion concentrations (blue triangles). Inclusion of 1 mM $MgCl_2$ slightly weakened the affinity of ATP-N for gelsolin (magenta circles) at 100 mM and 120 mM KCl (K, potassium ions, KM, potassium and magnesium ions).
