# Supplementary material for: ATP competes with PIP2 for binding to gelsolin
Source: PLoS One. 2018 Aug 7;13(8):e0201826. doi: 10.1371/journal.pone.0201826 (PMC6080781; doi:10.1371/journal.pone.0201826)
Supplement: S2 Fig — (A) The calcium-dependent gelsolin binding affinity for ATP under actin polymerization conditions was determined by monitoring the change in ATP-N anisotropy on gelsolin titration at different calcium ion concentrations. Each data point arises from the Kd calculated from a titration similar to Fig 1A. Kds were calculated to be between 1.51 μM and 2.35 μM in the 1 nM to 10 μM calcium concentration range, and the value increased to 8.05 ± 1.32 μM above 100 μM calcium. (B) The anisotropy of ATP-N (0.5 μM) bound to gelsolin (5 μM) was measured upon titrating with unlabeled ATP in the absence of calcium. 50 μM ATP was sufficient to remove the gelsolin bound ATP-N. The affinity of unlabeled ATP for gelsolin, under actin polymerizing salt conditions, was calculated to be Kd = 2.2 ± 0.17 μM measured by labeled/unlabeled ATP competition on gelsolin, which is similar to the affinity of ATP-N derived from Panel c in S1 Fig (Kd = 1.58 ± 0.8 μM). Data were analyzed by the method of Kubala (Kubala et al. 2004) with the modification that the change in anisotropy of ATP-N was substituted for the change in fluorescence emission as the indication of ATP competition. (PDF) [file pone.0201826.s002.pdf]

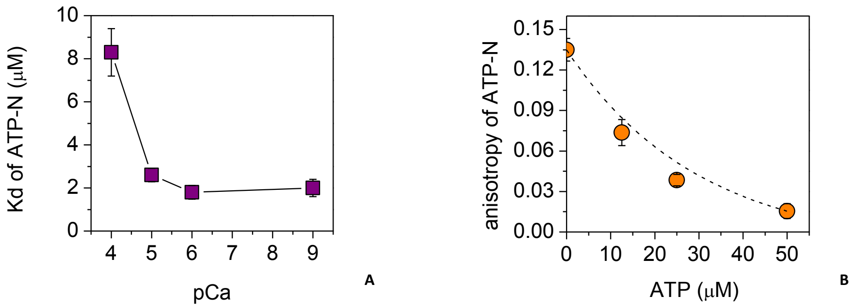

Fig. S2. ATP binding to gelsolin under actin polymerization conditions. (A) The calcium-dependent gelsolin binding affinity for ATP under actin polymerization conditions was determined by monitoring the change in ATP-N anisotropy on gelsolin titration at different calcium ion concentrations. Each data point arises from the K*d* calculated from a titration similar to Fig 1*a*. K*d*s were calculated to be between 1.51 μM and 2.35 μM in the 1 nM to 10 μM calcium concentration range, and the value increased to 8.05 ± 1.32 μM above 100 μM calcium. (B) The anisotropy of ATP-N (0.5 μM) bound to gelsolin (5 μM) was measured upon titrating with unlabeled ATP in the absence of calcium. 50 μM ATP was sufficient to remove the gelsolin bound ATP-N. The affinity of unlabeled ATP for gelsolin, under actin polymerizing salt conditions, was calculated to be K*d* = 2.2 ± 0.17 μM measured by labeled/unlabeled ATP competition on gelsolin, which is similar to the affinity of ATP-N derived from Fig. S1C (K*d* = 1.58 ± 0.8 μM). Data were analyzed by the method of Kubala (Kubala et al., 2004) with the modification that the change in anisotropy of ATP-N was substituted for the change in fluorescence emission as the indication of ATP competition.

References

Kubala, M., Plasek, J. and Amler, E. (2004). Fluorescence competition assay for the assessment of ATP binding to an isolated domain of Na+, K(+)-ATPase. *Physiol. Res.* **53**, 109-13.
