# Supplementary material for: ATP competes with PIP2 for binding to gelsolin
Source: PLoS One. 2018 Aug 7;13(8):e0201826. doi: 10.1371/journal.pone.0201826 (PMC6080781; doi:10.1371/journal.pone.0201826)
Supplement: S3 Fig — In the absence of divalent cations, the anisotropy of PIP2-F (0.5 μM) increased with increasing gelsolin concentration and this interaction was potassium ion dependent. The Kds calculated from the binding curves fitted with Eq 2 showed diminishing affinities with increasing potassium ion concentrations (blue triangles). Inclusion of 1 mM MgCl2 slightly weakened the affinity of PIP2-F for gelsolin (magenta squares) at 100 mM and 120 mM KCl (K, potassium ions, KM, potassium and magnesium ions). (B) Anisotropy of PIP2-F (0.5 μM) in the presence of gelsolin (5 μM) as a function of KCl or NaCl concentration. Data were fitted with simple sigmoidal curves. The decrease in anisotropy indicates that PIP2-F is dissociated from gelsolin by increasing potassium or sodium ion concentrations, with the half-effective concentrations of 141.2 ± 3.0 mM and 143.3 ± 1.6 mM, respectively. This indicates the effect of KCl on gelsolin:PIP2-F binding is largely ionic and nonspecific. (PDF) [file pone.0201826.s003.pdf]

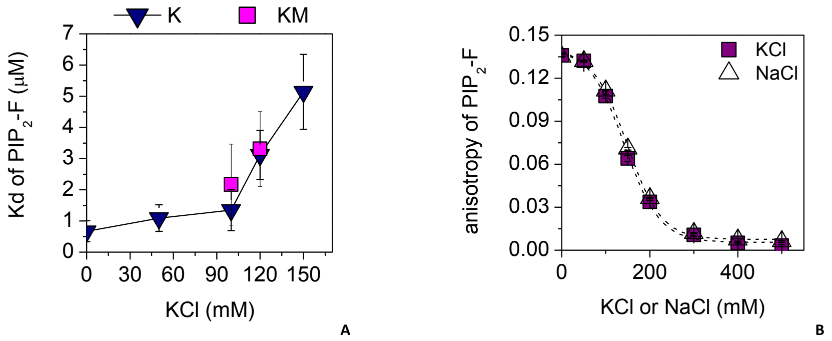

Fig. S3. The interplay between calcium ions, magnesium ions and ionic strength on PIP_2-F binding to gelsolin. In the absence of divalent cations, the anisotropy of PIP_2-F (0.5 μM) increased with increasing gelsolin concentration and this interaction was potassium ion dependent. The K*d*s calculated from the binding curves fitted with Eq. 2 showed diminishing affinities with increasing potassium ion concentrations (blue triangles). Inclusion of 1 mM $MgCl_2$ slightly weakened the affinity of PIP_2-F for gelsolin (magenta squares) at 100 mM and 120 mM KCl (K, potassium ions, KM, potassium and magnesium ions). (B) Anisotropy of PIP_2-F (0.5 μM) in the presence of gelsolin (5 μM) as a function of KCl or NaCl concentration. Data were fitted with simple sigmoidal curves. The decrease in anisotropy indicates that PIP_2-F is dissociated from gelsolin by increasing potassium or sodium ion concentrations, with the half-effective concentrations of 141.2 ± 3.0 mM and 143.3 ± 1.6 mM, respectively. This indicates the effect of KCl on gelsolin:PIP_2-F binding is largely ionic and nonspecific.
