# Supplementary material for: ATP competes with PIP2 for binding to gelsolin
Source: PLoS One. 2018 Aug 7;13(8):e0201826. doi: 10.1371/journal.pone.0201826 (PMC6080781; doi:10.1371/journal.pone.0201826)
Supplement: S4 Fig — (A) Determination of the actual concentrations of PIP2-F after incubation with different cations by light absorbance at 494 nm. T, 2 mM Tris-HCl, pH 7.4; K, 100 mM KCl; C, 1 mM CaCl2; M, 1 mM MgCl2. (B) Determination of critical micelle concentrations of PIP2 and PIP2-F (Panel inset) by dynamic light scattering. (PDF) [file pone.0201826.s004.pdf]

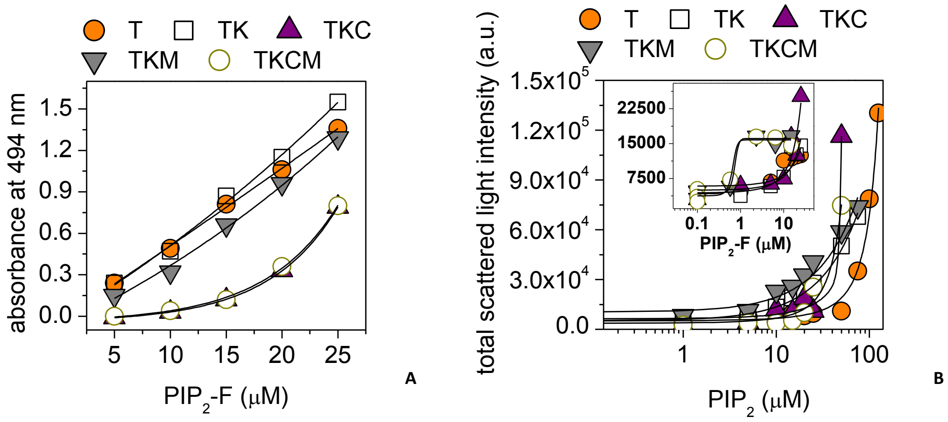

Fig. S4. Assessment of the solubility of PIP_2 and PIP_2-F in the experimental buffers. (A) Determination of the actual concentrations of PIP_2-F after incubation with different cations by light absorbance at 494 nm. T, 2 mM Tris-HCl, pH 7.4; K, 100 mM KCl; C, 1 mM $CaCl_2$; M, 1 mM $MgCl_2$. (B) Determination of critical micelle concentrations of PIP_2 and PIP_2-F (inset) by dynamic light scattering.

It is well known that divalent cations, calcium in particular, cause PIP_2 to aggregate (Flanagan et al., 1997; Levental et al., 2009). Therefore, we evaluated the effects of cations on the critical micelle concentrations (CMCs) of PIP_2 and PIP_2-F by dynamic light scattering (Huang et al., 2011). The results indicate clearly that the CMCs are affected by the cations. Calcium, but not magnesium or potassium ions, precipitated PIP_2-F, causing a substantial amount of PIP_2-F being removed from solution by centrifugation (Fig S4 *a*). After taking the changes in concentrations into account, the CMC of PIP_2-F was estimated to be ~10 μM in the absence of calcium, and this value was lowered to ~ 0.5 μM in the presence of 1 mM $CaCl_2$, either in the presence of 100 mM KCl or 100 mM KCl and 1mM $MgCl_2$ (Fig S4 *b*). These values are in agreement with those reported in the literature (Huang et al., 2011). Unlike PIP_2-F, the actual concentrations of PIP_2 could not be determined photometrically. Therefore, the CMCs of PIP_2 reported here were estimated based on the nominal rather than the actual PIP_2 concentrations. The obtained values were 20 μM in the absence of cations, 2 μM and < 1 μM in the presence of 100 mM KCl or100 mM KCl and 1 mM $MgCl_2$, respectively, which again are in concordance with the literature data (Flanagan et al., 1997). Introduction of calcium into the buffer raised the CMC of PIP_2 to ~ 10 μM, which is considered as an overestimation due to likely precipitation of PIP_2 by calcium in a similar way to that observed for PIP_2-F (Fig S4 *a*). Taken together, under the buffer conditions

tested, PIP_2-F was free from micelle formation at concentrations of 0.5 μM or below, whereas PIP_2 was more sensitive to cations and had a greater tendency to form micelles than PIP_2-F.

Methods: The critical micelle concentrations of PIP_2 and 1-(1-octadecanoyl-fluorescein-2R-octadecanoylphosphatidyl)inositol-4,5-bisphosphate (Cayman Chemical, abbreviated as PIP_2-F) were determined by dynamic light scattering (Huang et al., 2011). Fifty microliter samples were prepared by serial dilution of PIP_2 or PIP_2-F into a low salt buffer (2 mM Tris-HCl, pH 7.4), or the same buffer supplemented with 100 mM KCl, 1 mM $MgCl_2$, and/or 1 mM $CaCl_2$. After incubation for 30 min, the samples were centrifuged at 15,000 x g for 10 min, transferred into a 384-well clear bottom plate, and light scattering was measured using a Wyatt Dynapro plate reader at room temperature. The concentrations of PIP_2-F were subsequently determined by light absorbance at 494 nm with a Nanodrop spectrophotometer.

References

Flanagan, L. A., Cunningham, C. C., Chen, J., Prestwich, G. D., Kosik, K. S. and Janmey, P. A. (1997). The structure of divalent cation-induced aggregates of PIP2 and their alteration by gelsolin and tau. *Biophys J* **73**, 1440-1447.

Huang, W., Jiang, D., Wang, X., Wang, K., Sims, C. E., Allbritton, N. L. and Zhang, Q. (2011). Kinetic Analysis of PI3K Reactions with Fluorescent PIP2 Derivatives. *Anal Bioanal Chem* **401**, 1881-1888.

Levental, I., Christian, D. A., Wang, Y.-H., Madara, J. J., Discher, D. E. and Janmey, P. A. (2009). Calcium-dependent lateral organization in phosphatidylinositol 4,5-bisphosphate (PIP2)- and cholesterol-containing monolayers. *Biochemistry* **48**, 8241-8248.
