# Supplementary material for: ATP competes with PIP2 for binding to gelsolin
Source: PLoS One. 2018 Aug 7;13(8):e0201826. doi: 10.1371/journal.pone.0201826 (PMC6080781; doi:10.1371/journal.pone.0201826)
Supplement: S5 Fig — (A) Kds were calculated from the fit data with Eq 2 of gelsolin:PIP2-F binding under different calcium concentrations in the absence or presence of ATP. Part of the data have been shown in Fig 1D (white triangles). In the absence of ATP, PIP2-F (0.5 μM) binds to gelsolin, as reflected by thehigh affinity, across a wide range of calcium concentrations. In the presence of ATP, the interaction of PIP2-F with gelsolin was very weak below 10 μM calcium (Kd = 563.0 ± 3.6 μM at 10 μM calcium). (B) In the absence of gelsolin, calcium directly precipitates ATP-N (C, open triangles) with a half-maximum value of 70.4 ± 0.7 μM. Magnesium has no effect on the fluorescence emission of ATP-N either in the absence (M, orange circles) or presence of 10 μM calcium (MC, purple squares). Data were fitted with simple sigmoidal curves. (C) The reported FURA-2FF dissociation constant for calcium ions is 25 μM (A.G. Scientific Inc.). The calcium-dependent fluorescence emission profile of FURA-2FF (2 μM) over pCa range of 5–7 was similar in the presence and absence of 20 μM PIP2, which were fitted with simple sigmoidal curves. The half-saturation pCa values were = 6.265 ± 0.004 and 6.217 ± 0.011 in the absence and presence of PIP2, respectively, suggesting that PIP2 does not interact with calcium with a Kd less than 25 μM. The measurement was carried out with a Perkin Elmer LS-55 spectrofluorimeter (λex = 340 nm, λem = 505 nm). (PDF) [file pone.0201826.s005.pdf]

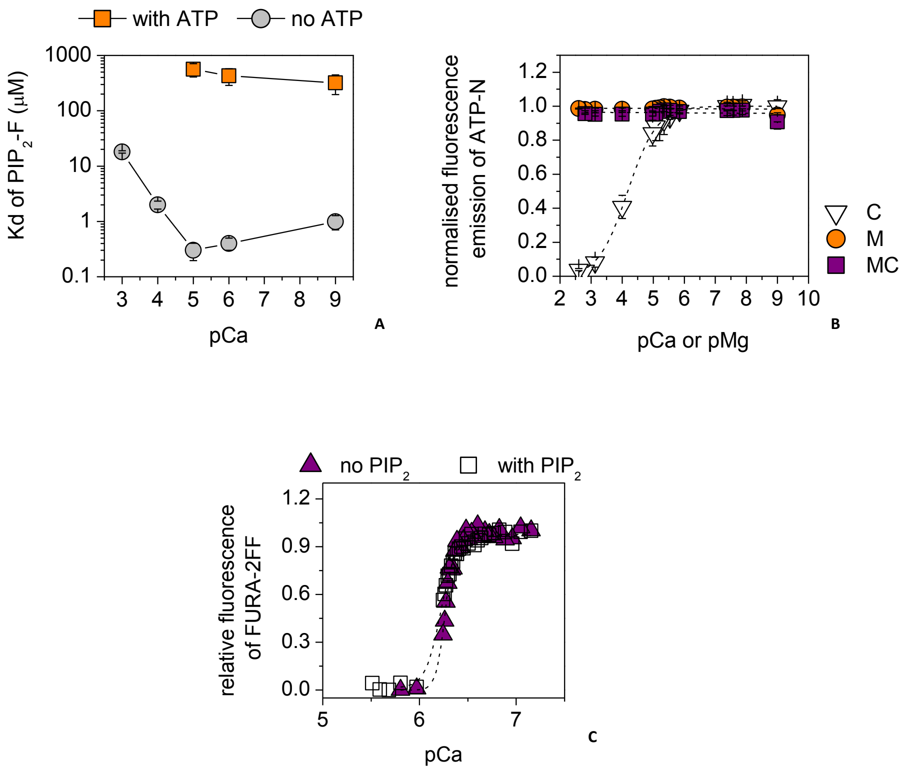

Fig. S5. Assessment of the effects of calcium and ATP on the binding of ATP-N and PIP_2-F to gelsolin. (A) K*d*s were calculated from the fit data with Eq.2 of gelsolin:PIP_2-F binding under different calcium concentrations in the absence or presence of ATP. Part of the data have been shown in Fig 1*d* (white triangles). In the absence of ATP, PIP_2-F (0.5 μM) binds to gelsolin, as reflected by thehigh affinity, across a wide range of calcium concentrations. In the presence of ATP, the interaction of PIP_2-F with gelsolin was very weak below 10 μM calcium (K*d* = 563.0 ± 3.6 μM at 10 μM calcium). (B) In the absence of gelsolin, calcium directly precipitates ATP-N (C, open triangles) with a half-maximum value of 70.4 ± 0.7 μM. Magnesium has no effect on the fluorescence emission of ATP-N either in the absence (M, orange circles) or presence of 10 μM calcium (MC, purple squares). Data were fitted with simple sigmoidal curves. (C) The reported FURA-2FF dissociation constant for calcium ions is 25 μM (A.G. Scientific Inc.). The calcium-dependent fluorescence emission profile of FURA-2FF (2 μM) over pCa range of 5-7 was similar in the presence and absence of 20 μM PIP_2, which were fitted with simple sigmoidal curves. The half-saturation pCa values were = 6.265 ± 0.004 and 6.217 ± 0.011 in the absence and presence of PIP_2, respectively, suggesting that PIP_2 does not interact with calcium with a K*d*

less than 25 μM. The measurement was carried out with a Perkin Elmer LS-55 spectrofluorimeter (λ_{ex} = 340 nm, λ_{em} = 505 nm).
