# Supplementary material for: ATP competes with PIP2 for binding to gelsolin
Source: PLoS One. 2018 Aug 7;13(8):e0201826. doi: 10.1371/journal.pone.0201826 (PMC6080781; doi:10.1371/journal.pone.0201826)
Supplement: S6 Fig — (A) Profiles of gelsolin-Alexa488 (red) and PIP2-containing vesicles filled with rhodamine590 (blue) in the absence of ATP. (B) Gelsolin-Alexa488 (red) was released and the size of phospholipid vesicle (filled by rhodamine590, blue) was changed by 0.5 mM ATP treatment in Fig 3B. (PDF) [file pone.0201826.s006.pdf]

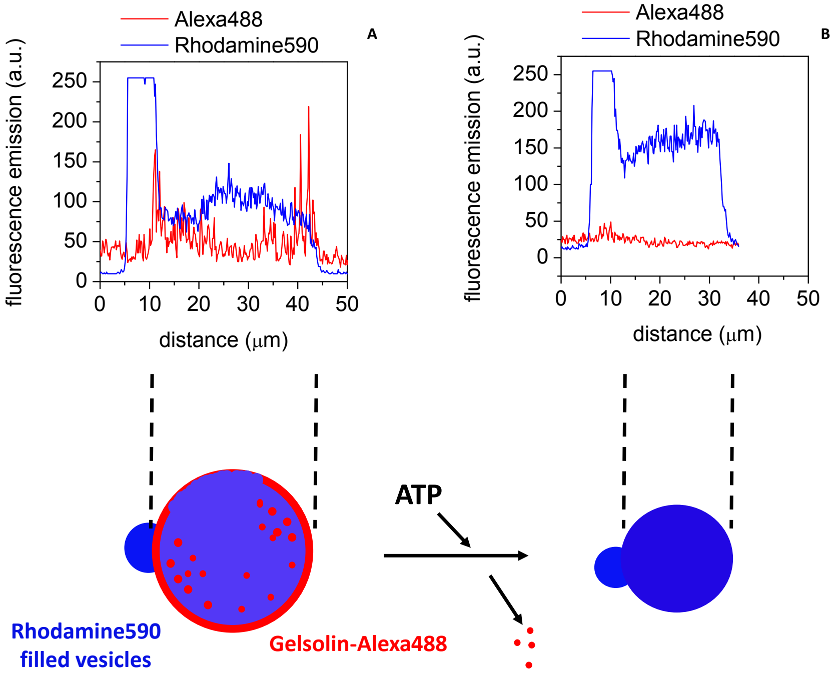

Fig. S6. Line scans of fluorescence intensity versus distance along the arrows shown in Fig 3*b*. (A) Profiles of gelsolin-Alexa488 (red) and PIP_2-containing vesicles filled with rhodamine590 (blue) in the absence of ATP. (B) Gelsolin-Alexa488 (red) was released and the size of phospholipid vesicle (filled by rhodamine590, blue) was changed by 0.5 mM ATP treatment in Fig 3*b*.
